# AI-Based Polymer Classification Using Ensemble Deep Learning and Heuristic Optimization: Implications for Recycling Applications

**DOI:** 10.3390/polym18101208

**Published:** 2026-05-15

**Authors:** Mohammad Anwar Parvez

**Affiliations:** Department of Chemical Engineering, College of Engineering, King Faisal University, P.O. Box 380, Al-Ahsa 31982, Saudi Arabia; mparvez@kfu.edu.sa

**Keywords:** artificial intelligence, polymers, ensemble of deep learning, heuristic search algorithm, feature selection, data pre-processing

## Abstract

Polymer-based product use is rapidly increasing worldwide, resulting in critical social, environmental, ecological, economic, and health effects. Worldwide efforts have increasingly focused on solutions to the equilibrium consumption, production, and disposal of plastics to tackle these issues. The frontiers of biodegradable and bio-based polymers are continually advancing in pursuit of sustainability. Therefore, designing ecological bioplastics made of both biodegradable and bio-based polymers reveals chances to overcome plastic pollution and resource depletion. Polymeric materials are mainly used to manufacture different products at the beginning of their lifespans and which become waste after usage. Numerous sustainability strategies and polymer recycling methods are described and mostly classified into chemical, mechanical, and thermal recycling processes. This manuscript presents a New Polymers Frontier in Recycling and Sustainability Using an Ensemble of Deep Learning with a Heuristic Search Algorithm (NPFRS-EDLHSA). This work is devoted to computational polymer typology, which is based on machine learning algorithms applied to data on physicochemical properties. Although polymer classification can facilitate downstream materials research, the present study does not directly simulate recycling, environmental impacts, or sustainability. The main contributions made by this work include (i) an exploratory analysis of ensemble deep learning models to classify polymers by type on a small and unbalanced dataset; (ii) an evaluation of the effect of feature selection with a heuristic optimization methodology; and (iii) a comparison of the effects on classification performance under limited data conditions. This research sets out to provide a methodological explanation, not arguments for industrial-scale applicability. For the polymer-type classification process, the proposed NPFRS-EDLHSA model designs an ensemble of deep learning techniques, namely a bidirectional recurrent neural network (BiRNN) model, a bidirectional gated recurrent unit (BiGRU) method, and a graph autoencoder (GAE) technique. Finally, the grasshopper optimization algorithm (GOA) adjusts the hyperparameter values of the ensemble models optimally and results in an improved classification performance. A wide-ranging set of experiments was conducted to validate the performance of the NPFRS-EDLHSA method. The experimental results indicated that the NPFRS-EDLHSA technique achieved a better performance than an existing model.

## 1. Introduction

Polymeric materials are essential in the modern world because of their durability and high strength-to-weight ratio [1]. In recent years, the production and use of plastics have expanded to include many other man-made materials, such as steel and cement, and millions of tons of plastics are produced worldwide. This number is expected to rise exponentially over the next 30 years across several production sectors [2]. Consequently, the rapid development of plastics has had a negative global effect stemming from the linear economy strategy, in which raw materials are gathered, converted into products, and ultimately discarded as waste [3]. Furthermore, the majority of plastic production utilizes non-renewable natural resources, and the handling of post-consumer products results in waste accumulation, environmental plastic contamination, and the depletion of additional resources. To address this issue, there is a need to create a more circular, closed-loop plastics consumption method. Plastic items and polymers owe their dominance and significant dependence to their remarkable physicochemical properties that guarantee affordability, durability, and light weight [4]. Due to their versatility, plastics are widely used materials with applications across multiple industrial sectors, like automotive vehicles, packaging, electronic gadgets, and construction [5].

Nowadays, recycling is the most significant activity available to decrease these effects and represents a popular and dynamic field in the plastics sector [6]. Reusing provides opportunities to decrease carbon dioxide emissions, the utilization of oil, and the quantities of waste requiring disposal [7]. Polymers are employed for many applications, including automotive, aerospace, sports, consumer goods, and medical; nevertheless, being non-biodegradable by nature, they extensively pollute the environment [8]. The waste disposal industries gather the material in general waste or in separated form, based on the geographical position [9]. Discharging waste into the environment and landfilling have led to enormous difficulties and pose a serious threat to life on Earth. Furthermore, raw material utilization and extraction might be decreased [10]. The application of artificial intelligence in polymer science is growing, but there are several problems to overcome. First, there is a limited availability of well-curated and labeled polymer datasets for the development and generalization of data-driven models. Datasets are often small, skewed, or missing standardized physicochemical descriptors, limiting their use. Second, it is inherently difficult to establish reliable relationships between polymer structure, physicochemical properties, and classification results: these relationships are complex and nonlinear due to complex molecular interactions. This makes it difficult for traditional machine learning approaches to identify important features. Third, although techniques such as deep learning or heuristic search have been used, there is a need for multi-strategy approaches in polymer informatics that can make use of multiple ways of feature engineering, machine learning, and optimization. Addressing these issues is essential to building intelligent systems for polymer classification, which are required to aid in downstream tasks such as material selection, recycling, and sustainable design.

This manuscript presents a New Polymers Frontier in Recycling and Sustainability Using an Ensemble of Deep Learning with Heuristic Search Algorithm (NPFRS-EDLHSA). The proposed NPFRS-EDLHSA model mainly focuses on providing an attractive solution for polymer recycling and sustainability using state-of-the-art techniques. The min–max normalization method is used in the pre-processing step to transform the feature values to a desired range, often [0, 1]. It allows all input variables to contribute equally to the model training and avoids features with large ranges from having more influence on the training process. It is worth noting that normalization does not eliminate noise from the data; rather, it scales the features and enhances numerical stability, especially in gradient-based optimization algorithms commonly employed in deep learning models. Following this, the binary dwarf mongoose optimizer (BDMO) algorithm has been implemented for the process of feature selection. For the polymer type classification process, the proposed NPFRS-EDLHSA model designs an ensemble of deep learning techniques, namely a bidirectional recurrent neural network (BiRNN) model, a bidirectional gated recurrent unit (BiGRU) method, and a graph autoencoder (GAE) technique. At last, the grasshopper optimization algorithm (GOA) adjusts the hyperparameter values of the ensemble models optimally and results in greater classification performance. A wide-ranging experimentation was performed to validate the performance of the NPFRS-EDLHSA method. The current research paper aims to compute the types of polymers by their physicochemical characteristics. Although polymer classification may be useful in materials research at the downstream level, this model does not explicitly simulate a recycling process or environmental impact.

## 2. Related Works

The integration of artificial intelligence (AI) into polymer recycling has gained significant attention in recent years, as the environmental and economic challenges posed by plastic waste become more pressing. Several studies have explored various AI techniques to enhance recycling processes, improve sorting accuracy, and optimize resource usage. Machine learning (ML) and deep learning (DL) methods have become central to addressing the challenges of polymer recycling. Kannan et al. [11] emphasize the application of ML and AI in multiple waste streams. These methods utilize AI-powered image detection and categorization to enhance distinct materials, assisting in improving the effectiveness of chemical recycling technologies; simultaneously, ML models allow cleaner processes for dealing with chemicals from material recovery. Higher accuracy is achieved in the elimination of beneficial elements from electronic waste through predictive analytics and automated disassembly. Despite the promising applications of AI, the recycling industry still faces significant hurdles. One such challenge is the inefficient classification of mixed plastic waste. Atasi et al. [12] proposed an AI-driven approach for developing innovative solutions in polymer synthesis and recycling processes. This model utilizes GA, which intends novel monomers and employs VFS to create nearly a million ROP polymers. ML techniques to forecast thermodynamic, thermal, and mechanical assets are vital for recyclability, and application-specific performance is employed to organize GA to optimize polymers. This model also projected possible additional polymers for polystyrene (PS) that attain each property, aiming to lower the estimated synthetic complexity. To overcome the limitations of traditional machine learning models, researchers have started integrating heuristic optimization techniques. The use of algorithms such as Genetic Algorithms (GAs), Particle Swarm Optimization (PSO), and Grasshopper Optimization Algorithm (GOA) has proven effective in improving the accuracy and efficiency of recycling processes. Duan et al. [13] elucidate the linkage between polymerization phenomena and catalyst deactivation in HG-AOP utilizing carbon materials. This technique shows that the removal of polymerization products results in a self-inhibition impact by sustaining a reliable polymerization energy barrier; however, to enhance the degree of polymerization (DP). These adherent layers contend with oxidants for active sites, obstructing electron transfer, impeding oxidant adsorption, and eventually hindering added catalytic activity.

Sundaralingam and Ramanathan [14] aimed to advance an automated method able to categorize plastic waste depending on its visual attributes. In real-world operation, an overhead camera places the positions of either the gripper or the waste items. By assessing the positional variance among gripper and waste, this method attains a higher degree of segregation precision, similar to human-like hand–eye coordination. Liang et al. [15] developed a method to incorporate the experimental process with AI to improve the purification of Se(IV)-affected water. In particular, an innovative approach is advanced for Se(IV) elimination by utilizing chitosan to decrease aqueous outputs of Se (IV).

Recent studies have shown that nanomaterial-reinforced polymer composites have become an important research direction because nanofillers can significantly modify the electrical, thermal, mechanical, and multifunctional behavior of polymeric materials. Carbon-based nanomaterials, particularly carbon nanotubes, graphene, graphene nanoplatelets, and carbon black, are widely investigated because their high aspect ratio, large surface area, and intrinsic conductivity allow them to form conductive networks inside polymer matrices. Shchegolkov et al. [16] reviewed the use of carbon nanotubes and graphene in polymer composites and highlighted the importance of synthesis, functionalization, and application strategies for improving functional composite performance. Similarly, Zare et al. [17] investigated the electrical conductivity of polymer composites containing carbon black nanoparticles and emphasized the roles of interphase depth, tunneling characteristics, and conductive network formation. From the thermal-property perspective, Li et al. [18] reviewed polymer-based thermally conductive functional materials and explained that suitable fillers can form thermally conductive networks or oriented structures within polymer matrices, thereby improving heat transfer performance. Musa et al. [19] further summarized recent advances in nano-enhanced polymer composite materials and showed that nanomaterials, especially carbon-based nanofillers, can improve electrical conductivity, thermal stability, mechanical strength, and multifunctional performance. In addition, VijayKashimatt et al. [20] reviewed conductive polymer composites with a focus on electrical conductivity and electromagnetic shielding, including conductivity mechanisms, thermal conductivity behavior, measurement techniques, and modeling approaches. These studies confirm that polymer properties are strongly influenced by nanofiller type, filler concentration, dispersion quality, polymer–filler interaction, interfacial characteristics, and conductive-network formation. However, unlike these studies, which mainly focus on experimental or theoretical analysis of nanofiller-enhanced electrical and thermal conductivity, the present work focuses on computational polymer typology classification using ensemble deep learning and heuristic optimization under limited and imbalanced data conditions.

Kern et al. [21] utilize digital reactions employing virtual forward synthesis (VFS) and ML models to quickly forecast mechanical, thermodynamic, and thermal assets. This work projected the structure, approaches, and primary outcome of the paper, emphasizing the possibility of ML and VFS. Lee et al. [22] focus on improving the rate of material recycling by advancing a separation method employing DL-based object detection. To enhance image data, labeling competence is attained for every type of material, and the execution of diverse labeling models is assessed. The average precision (AP) values for the single-material learning technique employing hybrid labeling showed better performance in contrast with other models. An integrated learning method is also advanced for composite materials. While significant progress has been made, many existing models still face challenges in terms of scalability, real-time processing, and environmental adaptability. Most approaches focus on isolated techniques, either applying deep learning or optimization algorithms, without fully exploring the synergies between these methods. The NPFRS-EDLHSA model presented in this study addresses these gaps by integrating deep learning techniques with heuristic search algorithms, providing a more robust, accurate, and scalable solution for polymer recycling and sustainability.

## 3. Proposed Method

**Figure 1:**
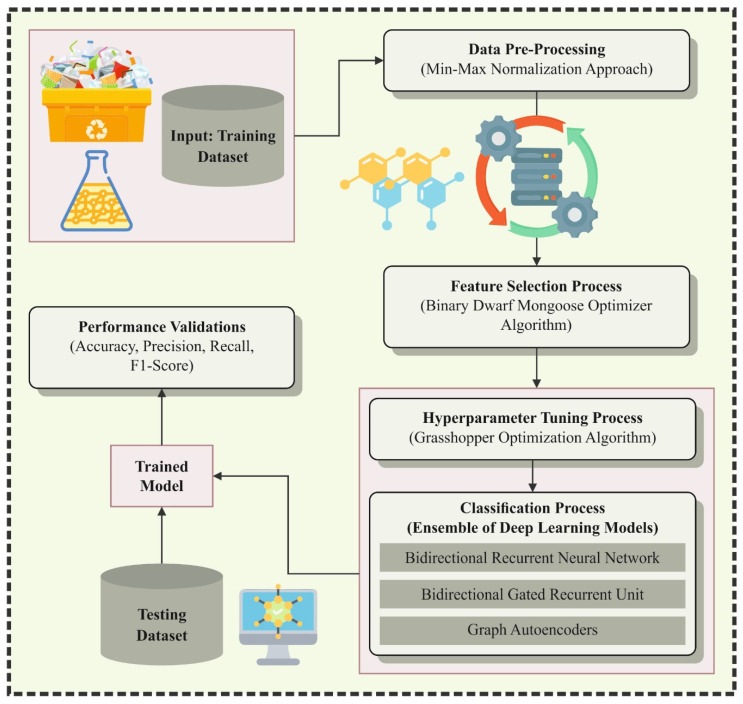
Overall flow of the NPFRS-EDLHSA method.

In this manuscript, we have presented an NPFRS-EDLHSA methodology. The proposed NPFRS-EDLHSA model mainly focuses on providing an attractive solution for polymer recycling and sustainability using state-of-the-art techniques. Figure 1 depicts the entire flow of the NPFRS-EDLHSA method.

### 3.1. Pre-Processing

The data pre-processing stage uses min–max normalization to scale the features as well as bring about numerical stability when training the models. This method normalizes each feature to an absolute range or [0, 1], depending on the lowest and highest values of the feature. There is a need to scale the features to ensure that attributes with higher numbers do not overpower the learning process, mainly through gradient-based optimization techniques. It is necessary to note that normalization does not eliminate noise in the data; on the contrary, it standardizes the magnitudes of features to ease the convergence and comparison of effective models across attributes. Initially, the min–max normalization is applied in the data pre-processing stage to clean and convert raw data into an appropriate format. Min–max normalization is an approach applied for scaling data inside a particular range, normally between 0 and 1, by converting the values according to the maximum and minimum values of the dataset [23]. In terms of sustainability and recycling of polymers, it is used to normalize different environmental indicators, like material efficiency, energy consumption, and waste reduction amounts, permitting simpler comparison through dissimilar polymer materials or recycling processes. By converting data to a steady range, this method assists in recognizing patterns and tendencies that are important in enhancing recycling methods. It furthermore helps the growth of methods that enhance resource sustainability and utilization efforts in polymer recycling. This method guarantees that changing data scales does not bias study or decision-making in sustainability advantages.

### 3.2. BDMO-Based Feature Selection

The BDMO algorithm has been implemented for the process of feature selection. The BDMO is derived from the constant variation that is DMO [24]. The model is influenced by the dwarf mongoose’s behavior and its migratory lifestyle, which frequently requires the transfer from one place to another in pursuit of food. Therefore, the mongoose’s location is the primary focus of the change or mutation in the optimizer procedure. In contrast to the BPSO and BEOSA, which utilize a few unique transfer functions for normalizing the animal’s population, the model depends on calculating the optimal candidate food location as exposed in Equation (1) and then states the mutation among 0 s and 1 s applied in $0.5<best_{i+1}^{fp}\leq 0.5$ Equation (2). $x_{i+1}^{fp}$ refers to the present optimal calculated food place, and the peep annotation is a sound produced in the search for this food locality. The calculation for the location of all DM within the population is obtained using Equation (3).

(1) $$best_{i+1}^{fp}=fp_{i}+phi\times peep$$

where phi represents the random scaling factor, and peep denotes a stochastic perturbation factor inspired by the communication behavior of dwarf mongooses during foraging.

(2) $$x^{d}=\left\{\begin{array}{l} selected\;feature\;x_{i+1}^{fp}>0.5\\ feature\;not\;selected\;x_{i+1}^{fp}\leq 0.5 \end{array}\right.$$

(3) $$x_{i+1}^{fp}=\left\{\begin{array}{l} best_{i}^{fp}-CF\times rand\times \left[x_{i}^{fp}-\vec{M}\right]\;if\;\varphi_{i+1}>\phi_{i}\;exploration\\ best_{i}^{fp}+CF\times rand\times \left[x_{i}^{fp}-\vec{M}\right]\;else\;exploitation \end{array}\right.$$

CF, which represents a parameter to direct the aggregate velocities of the mongooses’ group movement, in this equation, is inferred as $F={\left(1-\frac{iter}{{Max}_{iter}}\right)}^{\left(2\frac{iter}{{Max}_{iter}}\right)}$, and $\vec{M}$ is a vector monitoring the group movement to sleep in other sleeping mounds (sm), which can be signified by $\vec{M}=\sum_{i=1}^{n}\frac{x_{i}\times {sm}_{i}}{x_{i}}$. Here, ${Max}_{iter}$ and iter indicate the maximal iteration counts for the optimizer procedure and the present point of iteration within the optimizer procedure. Meanwhile, the φ annotation aids in calculating the average sm as specified by $\varphi =\sum_{i=1}^{n}\frac{sm_{i}}{n}$.

The fitness function (FF) reflects the classification precision and the selected feature counts. It maximizes the classification precision and reduces the set size of the selected features. As a result, the following FF is implemented to assess individual solutions, as presented in Equation (4).

(4) $$Fitness=\alpha \times ErrorRate+\left(1-\alpha \right)\times \frac{\#SF}{\#All\_F}$$

where ErrorRate represents the classification rate of error utilizing the designated features. #SF denotes selected feature counts and #All_F signifies total attribute counts in the new dataset. α has been applied to regulate the prominence of subset length and classification quality. In this test, α is set to 0.9.

### 3.3. Ensemble Classification Process

For the polymer type classification process, the proposed NPFRS-EDLHSA model designs an ensemble of deep learning models such as the BiRNN model, BiGRU method, and GAE technique.

#### 3.3.1. BiRNN Model

The entire sequence of features experiences an encoder utilizing a Bi-RNN network that stores the contextual vector for all output vectors [25]. The Bi-RNN’s forward and backward network iterate from time step t=1 to T and t=T to 1. This includes concurrent calculation of the forward and backward hidden sequences $h_{f}$ and $h_{b}$. The dual sequences are added to upgrade the sequence of output y, succeeding the patterns in Equations (5)–(7).

(5) $$h_{f}=W_{xh_{f}}x_{t}+W_{h_{f}}h_{f\left(t-1\right)}+b_{h_{f}}$$

(6) $$h_{b}=W_{xh_{b}}x_{t}+W_{h_{b}}h_{b\left(t-1\right)}+b_{h_{b}}$$

where $W_{xh_{f}}$ and $W_{xh_{b}}$ characterize the weighting matrices, which convert the input $x_{t}$ into the hidden layer (HL) area for the forward and reverse systems, respectively. $W_{h_{f}}$ and $W_{h_{b}}$ refer to weighted matrices, which link the HLs from the preceding time step to the present HL. The bias vectors $b_{h_{f}}$ and $b_{h_{b}}$ are added to the HLs to permit the method of learning an offset for improved data fitting.

At all time steps t, the forward and backward HLs, $h_{ft}$ and $h_{bt},$ are joined to yield the output sequence $y_{t},$ as demonstrated.

(7) $$y_{t}=W_{h_{f}}h_{ft}+W_{h_{b}}h_{bt}+b_{y}$$

**Figure 2:**
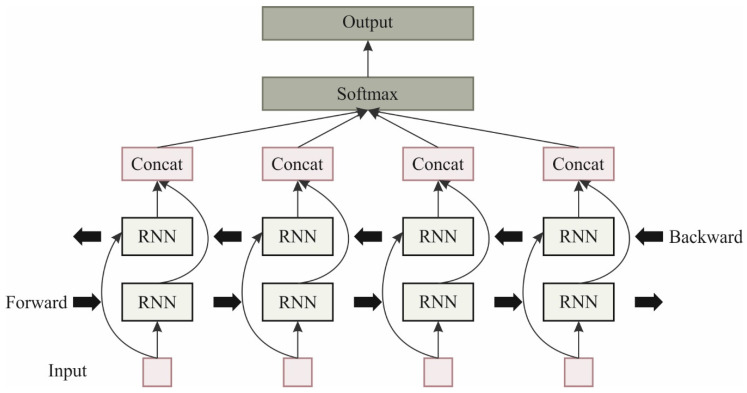
BiRNN structure.

$W_{h_{b}}$ and $Wh_{f}$ signify weighted matrices, which estimate the backward and forward HLs into the output area, and $b_{\gamma }$ represents the bias vector used for the output. The last output $y_{t}$ is therefore weighting mixtures of the backward and forward HLs, fine-tuned by the bias. Figure 2 illustrates the infrastructure of BiRNN.

#### 3.3.2. BiGRU Method

Bi-GRU is a multivariable time-series model derived from bidirectional GRU [26]. It may adjust to further composite sequence designs, and might successfully seize the dual-manner dependences in time-series data and the communication amongst numerous variables. One network processes time-series data from forward to reverse, while the other handles it from reverse to forward. In particular,

(8) $${\vec{h}}_{ft}=GRU\left({\vec{h}}_{f\left(t-1\right)},\;x_{t}\right)$$

(9) $${\vec{h}}_{bt}=GRU\left({\vec{h}}_{b\left(t+1\right)},\;x_{t}\right)$$

where ${\vec{h}}_{ft}$ and ${\vec{h}}_{bt}$ characterize the hidden layer (HL) from left to right and vice versa, correspondingly. GRU characterizes the GRU component, and $x_{t}$ embodies the tth component within the sequence of input. The last HL is the joining of HLs in dual directions.

(10) $$h_{t}=\left[{\vec{h}}_{ft};{\vec{h}}_{bt}\right]$$

Here, [;] signifies the joining process of vectors. At last, the HL is given to a fully connected (FC) layer to produce the result $y_{t}$. The model of $y_{t}$ is stated as

(11) $$y_{t}=softmax\left(Wh_{t}+b\right)$$

Now, b and W illustrate the bias and weight of the FC layer, correspondingly, and softmax signifies the activation function of SoftMax.

#### 3.3.3. GAE Classifier

The graph structure utilized in the research is the polymer samples, which are represented as the nodes, with the edge being defined by similarity in the normalized physicochemical feature space using a distance-based threshold. A graph autoencoder is used as an experimental representation learning aspect to determine whether relational statistics between samples can be used to improve classification results. Since the data is tabular, graph-based learning is used in an exploratory mode, and its usefulness is not presupposed but instead is assessed relative to each other. The effective application of graph data became a pressing concern, resulting in the need for investigators to explore the area of graph neural networks (GNNs) [27]. They proposed a new model for message aggregation and effectively utilized it in the conventional field of GNNs. Presently, the most commonly applied graph convolutional neural network (GCN) is the null field-based GCN. The theory of GCN originated from the model of the CNN in processing grid data, based on the basic concept of combining node-level data from localities to more effectively and flexibly gain the node’s feature representation. In conventional GCNs, the method of upgrading node features $H^{\left(l+1\right)}$ is characterized by Equation (12):

(12) $$H^{\left(l+1\right)}=\sigma ({\tilde{D}}^{-\frac{1}{2}}\tilde{A}{\tilde{D}}^{-\frac{1}{2}}H^{\left(l\right)})$$

where $\tilde{A}=A+I,I$ denotes the unit matrix, ${\tilde{D}}^{-\frac{1}{2}}$ refers to the normalization process of the matrix of degree $D,\;H^{\left(l\right)}$ stands for node feature representation at the present node layers by combining the characteristics of the nearby adjacent nodes, σ signifies a nonlinear activation function, and $H^{\left(l+1\right)}$ symbolizes node representation at the following layer.

GAE is the unsupervised learning method derived from GCN, tailored for aggregating important locality data and excavating the topological characteristics of graph structures. Nevertheless, conventional GCN models concentrate simply on particular local network architectures and require inadequate attention to higher-level data like node correlations and flow of information, leading to low performance in composite network studies. Still, these techniques concentrate only on sector-related strategies, regulating their flexibility and generalizability. Regarding the recent difficulties, this paper projects a universal incorporated algorithmic method; GAE accepts a multiview model to enlarge the receptive area of the model and makes a novel node embedding matrix by incorporating three dissimilar graph autoencoders to successfully remove important attributes. This combined mechanism is constructed to improve the universality of the model by removing main feature designs in some topologies of graph structures.

### 3.4. Parameter Tuning Using GOA

At last, the GOA adjusts the hyperparameter values of the ensemble models optimally, and the outcomes result in greater classification performance. The GOA is a bio-inspired swarm intelligence model, based on the collective and foraging swarming behavior detected in grasshoppers [28]. Extensively accepted by investigators, GOA has proved to be efficient in solving different optimization issues. Grasshoppers’ predatory strategies and unique social interactions, comprising constant location upgrades and encouraged configuration zones, allow them to balance local and global search, effectively recognizing the best solutions. The grasshopper swarming behavior is mathematically represented in Equation (13):

(13) $$P_{i}=S_{i}+G_{i}+A_{i}$$

where $P_{i}$ characterizes the position of the ith grasshopper, $S_{i}$ refers to the group behavior between grasshoppers within the swarm, $G_{i}$ specifies the gravitational force functioning on the ith grasshopper, and $A_{i}$ has been applied for modeling the influence of wind on the movement of the grasshopper. To combine this, the grasshopper’s random behavior can be restructured as (14):

(14) $$X_{i}=r_{1}S_{i}+r_{2}G_{i}+r_{3}A_{i}$$

where $r_{1},\;r_{2},$ and $r_{3}$ mean numbers dispersed in the interval of (0,1).

The description of social interaction Si is given as (15):

(15) $$S_{i}=\sum_{\begin{array}{c} j=1\\ j\neq 1 \end{array}}^{N}s\left(d_{ij}\right){\hat{d}}_{ij}$$

Here, $d_{ij}$ denotes the distance from the ith to the jth grasshopper, computed as dij=|xj−xi|. s has been applied to represent the social force intensity; ${\hat{d}}_{ij}$ signifies a unit vector originating from the ith to the jth grasshopper, measured as (16):

(16) $${\hat{d}}_{ij}=\frac{x_{j^{-x}i}}{d_{ij}}$$

The mathematical representation of social forces between grasshoppers, signified by the function s, was calculated as (17):

(17) $$s\left(r\right)=fe^{\frac{-r}{l}}-e^{-r}$$

Here, the variable f implies the attraction intensity, while l designates the scope of the captivating range. The group behavior between grasshoppers is defined by forces of repulsion and attraction. These are subject to the distance between the grasshoppers, which can be reflected in the interval of [0,15]. The force of gravity $G_{i}$ is computed as (18):

(18) $$G_{i}=-g\hat{e}$$

Now, the variable g characterizes the gravitational constant, and ${\hat{e}}_{g}$ means the unit vector focused on the center of the Earth.

Equation (19) displays the wind advection $A_{i}$:

(19) $$A_{i}=u{\hat{e}}_{w}$$

Here, the drift constant is u and the unit vector of the wind in the direction is ${\hat{e}}_{w}.$ By substituting the values of G and A, the resultant equation turns out to be

(20) $$X_{i}=\sum_{\begin{array}{c} j=1\\ j\neq 1 \end{array}}^{N}s\left(\left|x_{j}-x_{i}\right|\right)\frac{x_{j^{-x}i}}{d_{ij}}-g{\hat{e}}_{g}+u{\hat{e}}_{w}$$

Now, the total locust count within the population is N.

In Equation (20), it cannot be straightforwardly used for optimization functions owing to the particular restriction. During this expression, the grasshoppers quickly reach their secure position, which leads to the swarm’s incapacity to efficiently move towards the preferred target point. To deal with this problem, investigators have established an improved version of the equation, which can be given in Equation (21):

(21) $$X_{i}^{d}=c\left(\sum_{\begin{array}{c} j=1\\ j\neq 1 \end{array}}^{N}c\frac{ub_{d}-lb_{d}}{2}s\left(\left|x_{j}^{d}-x_{i}^{d}\right|\right)\frac{x_{j^{-x}i}}{d_{ij}}\right)+{\hat{T}}_{d}$$

Here, $lb_{d}$ and $ub_{d}$ specify the lower and upper limits in the dth dimension, correspondingly. ${\hat{T}}_{d}$ implies the best solution established to date inside the dth dimensional area, and c represents a control coefficient that regulates the balance between exploration and exploitation during the optimization process. The fitness choice is the extensive aspect that manipulates the performance of the GOA. The hyperparameter selection process comprises using the solution encoder model to compute the efficiency of candidate solutions. Also, the GOA utilizes precision as the primary criterion to define the fitness function (FF), as shown in the expression.

(22) $$Fitness=max\left(P\right)$$

(23) $$P=\frac{TP}{TP+FP}$$

Here, the true positive value symbolizes TP and the false positive value signifies FP.

To more comprehensively assess classification performance, other metrics, such as the Matthews Correlation Coefficient (MCC), are included alongside accuracy, precision, recall, and F1-score. MCC is suitable for imbalanced data as it takes into account true and false negatives and positives.

The MCC is defined as

(24) $$MCC=\frac{TP\times TN-FP\times FN}{\sqrt{\left(TP+FP)(TP+FN)(TN+FP)(TN+FN\right)}}$$

Moreover, to prevent data leakage and ensure the experiments’ validity, the data folder is partitioned using k-fold cross-validation. The data is split into training and testing sets using k-fold cross-validation to ensure that each fold is disjointed. Both feature selection and feature normalization are also performed within the training folds to avoid the model training being affected by the test set.

### 3.5. Polymer Data Representation and Feature Encoding

In the presented research, how the polymer data is represented is essential for the learning and classification process. A series of physicochemical properties is used to describe each polymer sample and is used as input features to the proposed model. The extracted features include important parameters such as glass transition temperature (Tg), melting point, viscosity, rheology, elasticity, Young’s modulus, and resilient modulus. These features are well-established as key characteristics that represent the thermal, mechanical, and structural behavior of polymers, thus offering valuable insights to distinguish among different groups of polymers. These features are tabulated as a vector format, with each polymer represented as a vector of features. These features are normalized before training to standardize the scales and enhance the numerical stability of the model during training. This format enables efficient processing of structured data by the deep learning models, as well as the interactions between multiple physicochemical features. To incorporate the graph-based aspect of the model, particularly the Graph Autoencoder (GAE), the tabular features are also converted into a graph. Here, polymer samples are represented as nodes, and edges are determined by similarity in the feature space after normalization. Using a distance threshold (e.g., Euclidean distance) to decide whether two nodes should be connected, an adjacency matrix representing the graph structure is created. This graph structure allows the model to learn latent relationships and neighborhood information among polymer samples, thereby enhancing the feature-based learning of the sequential models.

In summary, this bidirectional representation, which combines both tabular feature vectors and graph structures, allows for a more holistic approach in understanding polymer data by considering both individual property features and relationships among polymer samples.

## 4. Experimental Result and Analysis

The performance validation of the NPFRS-EDLHSA approach is verified under the dataset [29]. The computational implementation was carried out using Python, Python Software Foundation, Wilmington, DE, USA, available online: https://www.python.org/ (accessed on 14 December 2025). This dataset contains 42 instances under nine classes, as depicted in Table 1. An initial set of multiple physicochemical features is considered for polymer representation. Key parameters such as glass transition temperature (ranging from −70 °C to 150 °C), melting points (from 100 °C to 350 °C), viscosity (from 0.01 to 1000 Pa·s), rheology (between 0.1 and 10 Pa·s), elasticity (ranging from 1 to 10 GPa), Young’s modulus (ranging from 0.5 to 4 GPa), and resilient modulus (between 1 and 5 GPa) are integrated. These features provide critical insights into the physical properties and behavior of the polymers. By including these parameters, the model is better equipped to capture the associations among diverse polymer types, resulting in more accurate classification of the nine class labels. The inclusion of these chosen features significantly improves the capability of the technique to distinguish between classes and improve overall classification performance. The data used in this paper will be a sample of 42 polymer samples in nine polymer classes, where there is an imbalance of classes and very few samples on some classes. This is not a large enough dataset to train deep learning models that have a high guarantee of generalization. Therefore, the outcomes of the experiment are to be understood as exploratory and proof-of-concept instead of being statistically conclusive. This research is aimed at discussing methodological behavior in limited data conditions instead of creating an implementable classification system. Larger and publicly verifiable datasets with an equal content of classes will be necessary in the future to support the strong validation. In order to minimize bias due to a single train–test split, the evaluation of the models is conducted through the cross-validation with k-fold. The performance measures are reported in the form of an average within the folds. Class imbalance leads to precision, recall, and F1-score being evaluated together to prevent the interpretation of performance in a misguided way. The high scores in classifications that have been observed should be viewed with a lot of caution because small sample sizes tend to inflate performance estimates.

**Figure 3:**
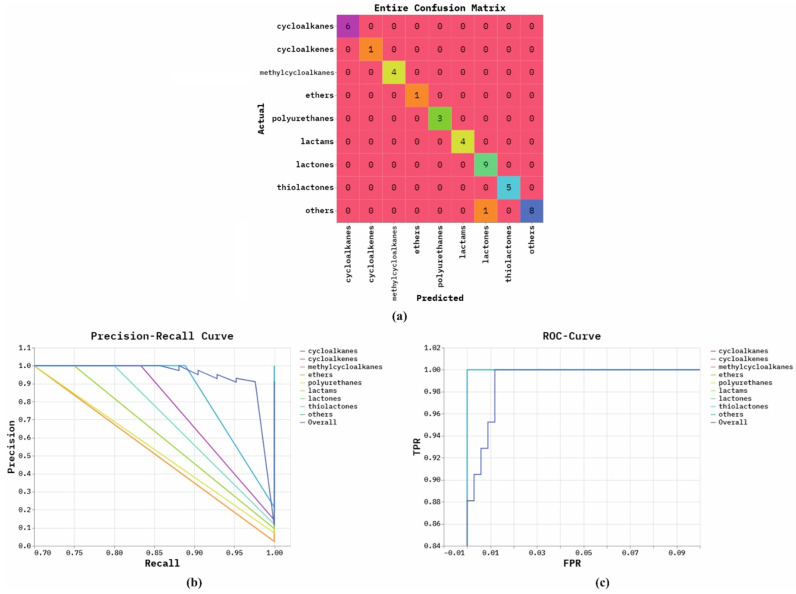
Classifier outcome of NPFRS-EDLHSA model: (**a**) entire confusion matrix, (**b**) curve of PR, and (**c**) curve of ROC.

Figure 3 shows the classifier performances of the NPFRS-EDLHSA model. Figure 3a represents the entire confusion matrix through the precise detection and classification of all classes. Figure 3b illustrates the PR study, which indicates an improved performance over all classes. Finally, Figure 3c demonstrates the ROC outcome, which signifies capable solutions with great ROC values for dissimilar class labels.

**Figure 4:**
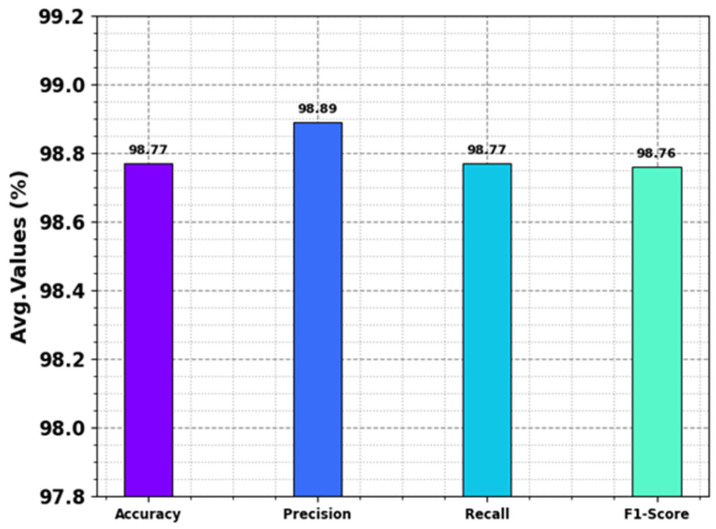
Average of NPFRS-EDLHSA approach under various metrics.

Table 2 and Figure 4 deliver the classifier result of the NPFRS-EDLHSA method below several metrics. The performances indicate that the NPFRS-EDLHSA method accurately detected and classified all the classes. The proposed NPFRS-EDLHSA algorithm obtains an average $accu_{y}$ of 98.77%, $prec_{n}$ of 98.89%, $reca_{l}$ of 98.77%, and $F1_{score}$ of 98.76%.

**Figure 5:**
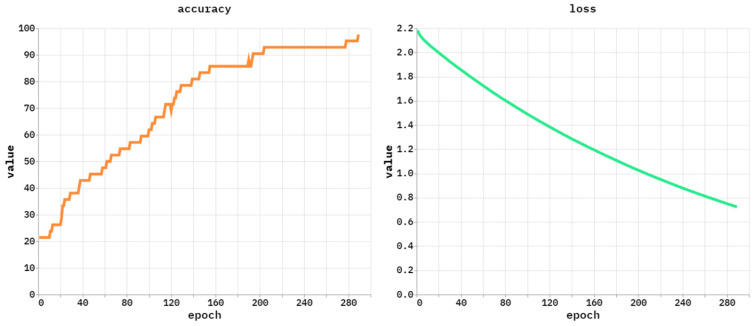
Accuracy and loss analysis of NPFRS-EDLHSA approach.

Figure 5 depicts the accuracy and loss study of the NPFRS-EDLHSA system. The accuracy outcome of the NPFRS-EDLHSA technique gains cumulative values across growing epochs. Moreover, the rising validation through training indicates that the NPFRS-EDLHSA model performs proficiently on the test dataset. At last, the loss curve implies that the NPFRS-EDLHSA algorithm attains closer values. The NPFRS-EDLHSA model understands the test dataset competently.

Table 3 offers a comparative study of the NPFRS-EDLHSA method with existing methodologies below several metrics [30].

**Figure 6:**
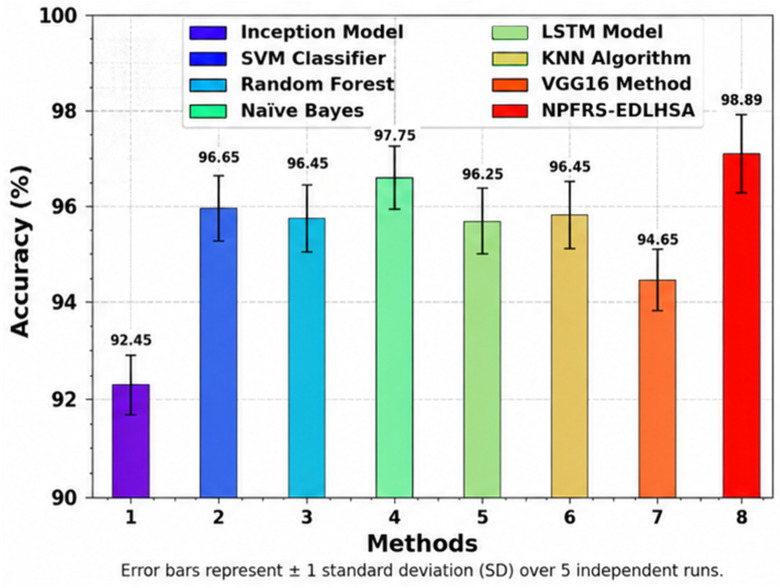
$Accu_{y}$ outcome of NPFRS-EDLHSA technique with existing models.

Figure 6 examines the $accu_{y}$ result of the NPFRS-EDLHSA model with existing approaches. The figure shows that the proposed NPFRS-EDLHSA technique has reached a greater $accu_{y}$ value of 98.77%. In the meantime, the existing algorithms, such as Inception, SVM, RF, NB, LSTM, KNN, and VGG16 models, have attained diminishing returns of $accu_{y}$ values of 92.54%, 96.70%, 96.53%, 97.79%, 96.28%, 96.52%, and 94.74%.

**Figure 7:**
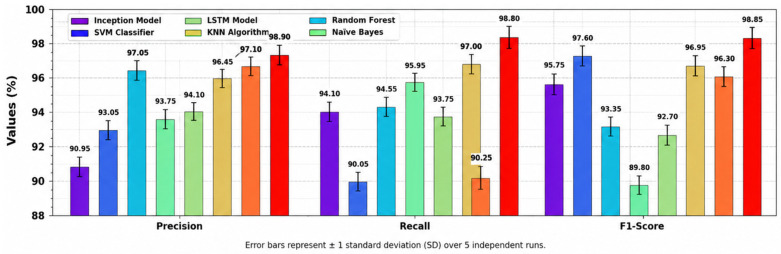
$Prec_{n}$, $reca_{l},$ and $F1_{score}$ outcome of NPFRS-EDLHSA algorithm with existing models.

Figure 7 studies the $prec_{n}$, $reca_{l},$ and $F1_{score}$ performance of the NPFRS-EDLHSA system with existing methodologies. According to $prec_{n},$ the NPFRS-EDLHSA model has a superior $prec_{n}$ of 98.89%, whereas the Inception, SVM, RF, NB, LSTM, KNN, and VGG16 algorithms have a minimum $prec_{n}$ of 90.99%, 92.98%, 96.99%, 93.74%, 94.14%, 96.44%, and 97.08%, correspondingly. Moreover, according to ${reca}_{l},$ the NPFRS-EDLHSA model has a superior ${reca}_{l}$ of 98.77%, whereas the Inception, SVM, RF, NB, LSTM, KNN, and VGG16 algorithms have a minimum ${reca}_{l}$ of 94.06%, 90.02%, 94.61%, 95.95%, 93.84%, 96.98%, and 90.25%, correspondingly. In addition, according to ${F1}_{score},$ the NPFRS-EDLHSA model has a superior ${F1}_{score}$ of 98.76%, whereas the Inception, SVM, RF, NB, LSTM, KNN, and VGG16 algorithms have a minimum ${F1}_{score}$ of 95.83%, 97.63%, 93.36%, 89.86%, 92.72%, 96.88%, and 96.28%, correspondingly.

**Figure 8:**
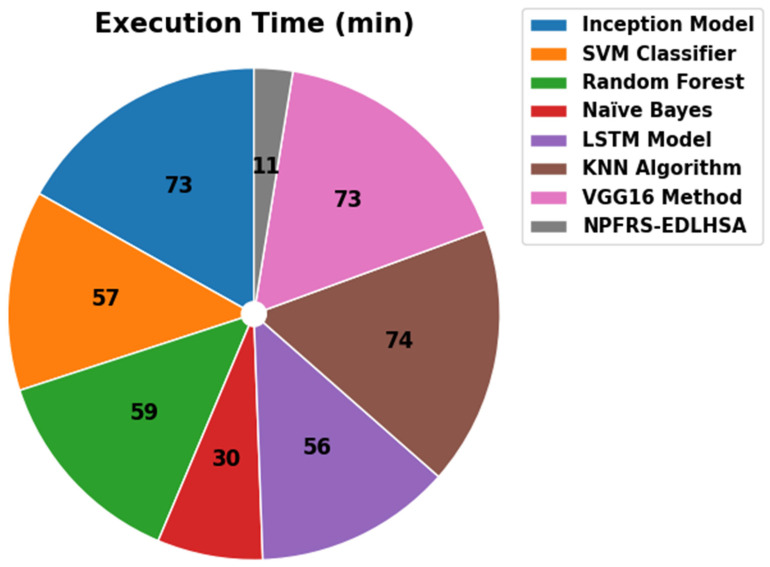
ET outcome of NPFRS-EDLHSA method with existing models.

In Table 4 and Figure 8, the execution time of the NPFRS-EDLHSA method with existing techniques is showcased. The performances imply that the NPFRS-EDLHSA algorithm produces a minimal ET value of 11 min, while the Inception, SVM, RF, NB, LSTM, KNN, and VGG16 models obtain better ET values of 73 min, 57 min, 59 min, 30 min, 56 min, 74 min, and 73 min, respectively. Benchmarks on execution time are documented with the same hardware and training settings, but the architectures of different models and their convergence characteristics differ, so they are only indicative and not absolute benchmarks.

The size of the dataset used in this work is small and is suitable only for initial explorations. Although the proposed model shows a good classification accuracy, it is worth noting that the small sample size is associated with an increased risk of overfitting, where the model may fit the training data but not capture general patterns. While k-fold cross-validation has been used to reduce this effect, it is impossible to completely rule out optimistic bias in the performance estimates due to the limited sample size and class imbalance. Moreover, the current research has not involved external validation on independent or large-scale polymer datasets, which is crucial to validate the performance and generalization of the proposed model. This limits the claim of general applicability of the method in polymer classification and recycling applications. Hence, the findings in this work should be regarded as proof-of-concept and, in future studies, the proposed model will be tested using larger datasets, publicly available data, and more diverse data to improve the reliability and reproducibility.

In addition to the numerical evaluation, the observed classification performance can be related to the underlying physicochemical behavior of polymers. The selected features, such as glass transition temperature, melting point, viscosity, and elastic modulus, are directly associated with molecular structure, chain mobility, and intermolecular interactions. For instance, polymers with higher glass transition temperatures and modulus values typically exhibit stronger intermolecular forces and restricted chain movement, making them structurally distinct from more flexible polymers. Similarly, variations in viscosity and rheological behavior reflect differences in molecular weight distribution and chain entanglement. The proposed model effectively captures these nonlinear relationships between structural properties and polymer types, which contributes to its improved classification performance. Thus, the results are not only computationally significant but also consistent with established principles of polymer chemistry and material behavior.

The ablation study is performed with the presence and absence of each level in the proposed model, and the performance is presented in Table 5.

To better understand the contribution of each component in the proposed NPFRS-EDLHSA model, an ablation study was carried out by removing key modules one at a time. It was observed that the performance gradually decreased whenever a component such as BiGRU, GAE, feature selection (BDMO), or hyperparameter tuning (GOA) was excluded. This clearly indicates that each part of the model contributes to improving the overall classification performance. In addition, a paired *t*-test was performed, and the results showed that the improvements achieved by the proposed model are statistically significant (*p* < 0.05). However, since the dataset used in this study is relatively small, these results should be interpreted within the context of the current experimental setup.

## 5. Conclusions

In this manuscript, we have presented an NPFRS-EDLHSA. The proposed NPFRS-EDLHSA model mainly focuses on providing an attractive solution for polymer recycling and sustainability using state-of-the-art techniques. Initially, the min–max normalization is applied in the data pre-processing stage for noise removal. Following that, the BDMO algorithm has been implemented for the process of feature selection. For the polymer type classification process, the proposed NPFRS-EDLHSA model designs an ensemble of deep learning models, such as the BiRNN model, BiGRU method, and GAE technique. At last, the GOA adjusts the hyperparameter values of the ensemble models optimally, and the outcomes result in greater classification performance. The model was tested experimentally and showed an average accuracy of 98.77 and precision, recall, and F1-score values of 98.89, 98.77, and 98.76, respectively. Although these findings indicate that the model is capable of differentiating the types of polymers in the dataset under investigation, it is important to recognize that the sample size used is rather small to make the results statistically sound. A wide-ranging experimentation was performed to validate the performance of the NPFRS-EDLHSA method. The experimental results specified that the NPFRS-EDLHSA technique emphasized betterment over another existing model. The next stage of this exploration framework is to incorporate larger polymer datasets by incorporating more explicit models of recyclability, degradability, and properties related to processing and use, which would allow for developing a more substantial relationship between polymer classification and sustainability-oriented decision-making.

## Figures and Tables

**Table 1:** Dataset specification.

Class	Sample Number
Cycloalkanes	6
Cycloalkenes	1
Methylcycloalkanes	4
Ethers	1
Polyurethanes	3
Lactams	4
Lactones	9
Thiolactones	5
Others	9
Total Instances	42

**Table 2:** Classifier output of NPFRS-EDLHSA technique under various metrics.

Class	$Accu_{y}$	$Prec_{n}$	$Reca_{l}$	${F1}_{score}$
Cycloalkanes	100.00	100.00	100.00	100.00
Cycloalkenes	100.00	100.00	100.00	100.00
Methylcycloalkanes	100.00	100.00	100.00	100.00
Ethers	100.00	100.00	100.00	100.00
Polyurethanes	100.00	100.00	100.00	100.00
Lactams	100.00	100.00	100.00	100.00
Lactones	100.00	90.00	100.00	94.74
Thiolactones	100.00	100.00	100.00	100.00
Others	88.89	100.00	88.89	94.12
Average	98.77	98.89	98.77	98.76

**Table 3:** Comparison evaluation of the NPFRS-EDLHSA model with existing techniques.

Methods	$Accu_{y}$	$Prec_{n}$	$Reca_{l}$	${F1}_{score}$
Inception Model	92.54	90.99	94.06	95.83
SVM Classifier	96.70	92.98	90.02	97.63
Random Forest	96.53	96.99	94.61	93.36
Naïve Bayes	97.79	93.74	95.95	89.86
LSTM Model	96.28	94.14	93.84	92.72
KNN Algorithm	96.52	96.44	96.98	96.88
VGG16 Method	94.74	97.08	90.25	96.28
NPFRS-EDLHSA	98.77	98.89	98.77	98.76

**Table 4:** Execution time comparison of the NPFRS-EDLHSA method and existing models.

Method	Execution Time (min)
Inception Model	73
SVM Classifier	57
Random Forest	59
Naïve Bayes	30
LSTM Model	56
KNN Algorithm	74
VGG16 Method	73
NPFRS-EDLHSA	11

**Table 5:** Ablation study and performance of the proposed model.

Model Variant	Accuracy (%)	Precision (%)	Recall (%)	F1-Score (%)
BiRNN Only	94.21	94.65	93.98	94.1
BiRNN + BiGRU	95.87	96.12	95.74	95.92
Without GAE	96.45	96.8	96.21	96.5
Without BDMO (Feature Selection)	96.92	97.1	96.75	96.88
Without GOA (Hyperparameter Tuning)	97.35	97.6	97.2	97.32
Proposed NPFRS-EDLHSA	98.77	98.89	98.77	98.76

## Data Availability

The original contributions presented in this study are included in the article. Further inquiries can be directed to the corresponding authors.
